# Ticagrelor or prasugrel vs. clopidogrel in patients with atrial fibrillation undergoing percutaneous coronary intervention for myocardial infarction

**DOI:** 10.1093/ehjopen/oead134

**Published:** 2023-12-14

**Authors:** Sissel J Godtfredsen, Kristian H Kragholm, Anna Meta Dyrvig Kristensen, Tarek Bekfani, Rikke Sørensen, Maurizio Sessa, Christian Torp-Pedersen, Deepak L Bhatt, Manan Pareek

**Affiliations:** Department of Cardiology, Aalborg University Hospital, Aalborg, Denmark; Department of Cardiology, Aalborg University Hospital, Aalborg, Denmark; Department of Cardiology, Copenhagen University Hospital—Bispebjerg and Frederiksberg, Copenhagen, Denmark; Department of Cardiology, Otto-von-Guericke-Universität Magdeburg, Magdeburg, Germany; Department of Cardiology, Copenhagen University Hospital—Rigshospitalet, Blegdamsvej 9, 2100 Copenhagen Ø, Denmark; Department of Drug Design and Pharmacology, University of Copenhagen, Copenhagen, Denmark; Department of Cardiology, Copenhagen University Hospital—North Zealand Hospital, Hillerød, Denmark; Mount Sinai Fuster Heart Hospital, Icahn School of Medicine at Mount Sinai, New York, NY, USA; Department of Cardiology, Copenhagen University Hospital—Rigshospitalet, Blegdamsvej 9, 2100 Copenhagen Ø, Denmark; Center for Translational Cardiology and Pragmatic Randomized Trials, Department of Cardiology, Copenhagen University Hospital—Herlev and Gentofte, Gentofte Hospitalsvej 8, 3. TH, 2900 Hellerup, Denmark

**Keywords:** Myocardial infarction, Atrial fibrillation, Triple antithrombotic therapy, Double antithrombotic therapy, P2Y_12_ inhibitor

## Abstract

**Aims:**

The efficacy and safety of ticagrelor or prasugrel vs. clopidogrel in patients with atrial fibrillation (AF) on oral anticoagulation (OAC) undergoing percutaneous coronary intervention (PCI) for myocardial infarction (MI) have not been established.

**Methods and results:**

This was a nationwide cohort study of patients on OAC for AF who underwent PCI for MI from 2011 through 2019 and were prescribed a P2Y_12_ inhibitor at discharge. The primary efficacy outcome was major adverse cardiovascular events (MACE), defined as a composite of death from any cause, stroke, recurrent MI, or repeat revascularization. The primary safety outcome was cerebral, gastrointestinal, or urogenital bleeding requiring hospitalization. Absolute and relative risks for outcomes at 1 year were calculated through multivariable logistic regression with average treatment effect modelling. Outcomes were standardized for the individual components of the CHA_2_DS_2_-VASc and HAS-BLED scores as well as type of OAC, aspirin, and proton pump inhibitor use. We included 2259 patients of whom 1918 (84.9%) were prescribed clopidogrel and 341 (15.1%) ticagrelor or prasugrel. The standardized risk of MACE was significantly lower in the ticagrelor or prasugrel group compared with the clopidogrel group (standardized absolute risk, 16.3% vs. 19.4%; relative risk, 0.84, 95% confidence interval, 0.70–0.98; *P* = 0.02), while the risk of bleeding did not differ (standardized absolute risk, 5.5% vs. 5.1%; relative risk, 1.07, 95% confidence interval, 0.73–1.41; *P* = 0.69).

**Conclusion:**

In patients with AF on OAC who underwent PCI for MI, treatment with ticagrelor or prasugrel vs. clopidogrel was associated with reduced ischaemic risk, without a concomitantly increased bleeding risk.

## Graphical Abstract

**Graphical abstract:**
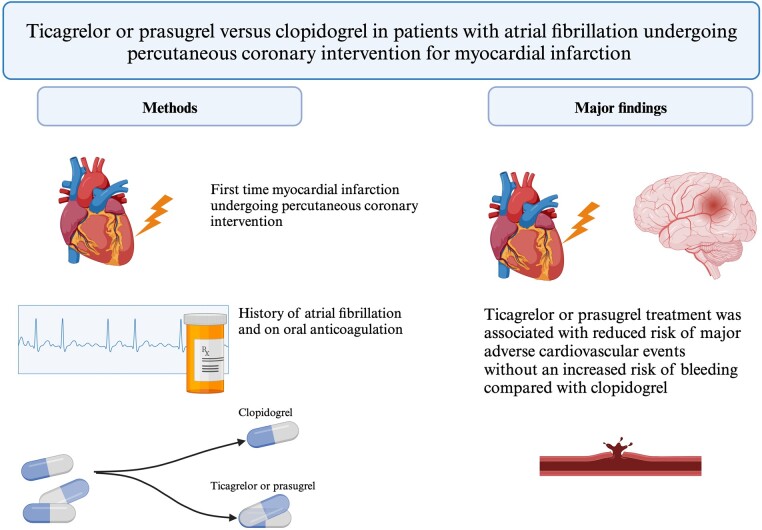

## Introduction

Coronary artery disease and atrial fibrillation (AF) frequently coexist, and the concomitant presence of both conditions is associated with an increased risk of adverse cardiovascular events.^1,2^ In addition, AF is an independent, modifiable risk factor for ischaemic stroke.^3^ Even though the AF population is heterogeneous in terms of annual stroke risk (ranging from 2 to 10%), more than 80% of patients with AF are treated with oral anticoagulant therapy (OAC).^4–8^ While the benefit of OAC for stroke prevention is clear, risk factors for stroke overlap with those for bleeding events, and bleeding risk is further increased by OAC.^9,10^

Twelve months of dual antiplatelet therapy with aspirin and a P2Y_12_ inhibitor is the recommended treatment in most patients with acute coronary syndromes (ACS).^11,12^ The randomized Platelet Inhibition and Platelet Outcomes (PLATO) and Therapeutic Outcomes by Optimizing Platelet Inhibition with Prasugrel-Thrombolysis in Myocardial Infarction (TRITON-TIMI 38) trials established the superiority of dual antiplatelet therapy with ticagrelor or prasugrel compared with clopidogrel, in reducing the risk of recurrent ischaemic events in patients with ACS.^13,14^ However, the greater potency of ticagrelor and prasugrel came at the expense of an increased bleeding risk, and both trials excluded patients in whom concomitant OAC was indicated. Moreover, the far majority of participants included in the randomized trials of double therapy (OAC plus P2Y_12_ inhibitor) or triple therapy (OAC plus dual antiplatelet therapy) in the setting of AF and percutaneous coronary intervention (PCI) for coronary artery disease received clopidogrel instead of one of the more potent P2Y_12_ inhibitors.^15–18^ As a result, contemporary guidelines recommend clopidogrel as the P2Y_12_ inhibitor of choice in patients with AF and ACS treated with PCI.^12^

Considering the limited amount of data in the field, we aimed to investigate the efficacy and safety of ticagrelor and prasugrel compared with clopidogrel in patients with a history of AF who were admitted with myocardial infarction (MI) and underwent PCI.

## Methods

### Study design and data sources

We performed a registry-based, nationwide study including data from (i) the Registry of Causes of Death, which holds information on death since 1970;^19^ (ii) the Danish National Prescription Registry, which contains information on all filled prescriptions since 1995 sorted by Anatomical Therapeutic Chemical (ATC) codes;^20^ and (iii) the Danish National Patient Registry (DNPR), which contains information on all hospital admissions, discharge diagnoses, and procedure codes using the International Classification of Diseases (ICD) system and the Nordic Medico Statistical Committee (NOMESCO) classification since 1978.^21^ The databases were linked on an individual level through an encrypted Civil Personal Registry (CPR) number on a Statistics Denmark server.

### Setting

**Figure 1:**
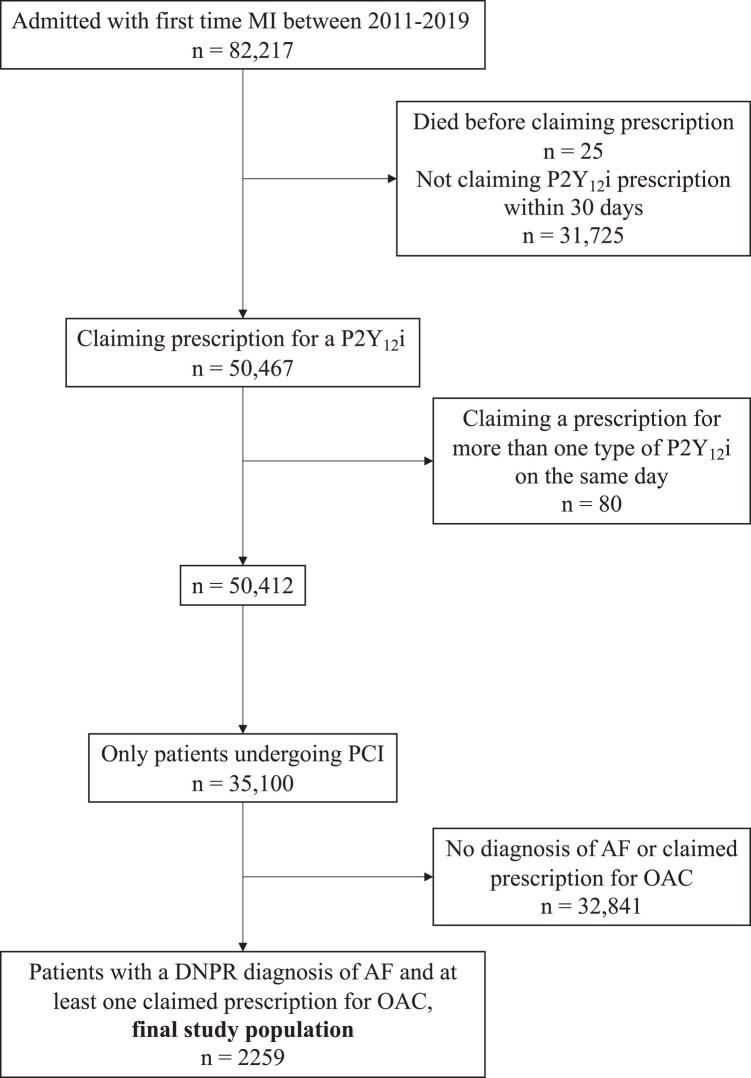
Flow chart illustrating selection of the study population. MI, myocardial infarction; P2Y_12_i, P2Y_12_ inhibitor; PCI, percutaneous coronary intervention; AF, atrial fibrillation; OAC, oral anticoagulation; DNPR, the Danish National Patient Registry.

We included patients at least 18 years of age who (i) had a diagnosis of atrial fibrillation and were treated with an OAC, (ii) were hospitalized for a first-time acute MI from 1 January 2011 through 31 December 2019, (iii) underwent PCI within 7 days of admission, and (iv) claimed a prescription for clopidogrel, ticagrelor, or prasugrel within 30 days of discharge. We excluded patients who were treated with a P2Y_12_ inhibitor prior to admission. *Figure 1* summarizes the study selection process. The index date was defined as the day of the first claimed prescription for a P2Y_12_ inhibitor.

We included comorbidities documented up to 10 years prior to index hospitalization using DNPR discharge and outpatient diagnoses. A list of included comorbidities and their definition by the ICD, Tenth Revision (ICD-10) codes, procedure codes, and/or ATC codes is included in Supplementary material online, *Table S1*. Diabetes mellitus and chronic obstructive pulmonary disease were defined as either DNPR diagnoses (up to 10 years prior) or through claim of an antidiabetic or either an anticholinergic or anticholinergic/antiadrenergic drug, respectively, within 180 days of index hospitalization. Hypertension was defined as either an ICD-10 diagnosis or by ≥2 claims of ≥2 antihypertensive drugs within two consecutive quarters no more than 5 years prior to index hospitalization.^22^

Claimed prescriptions of aspirin, proton pump inhibitors (PPI), and non-steroidal anti-inflammatory drugs (NSAID) ≤ 180 days of index hospitalization were tracked as well.

Proportion of days covered (PDC) was employed as a measure of adherence to P2Y_12_ inhibitor, direct oral anticoagulant (DOAC), and aspirin treatment, respectively.^23^ Proportion of days covered was defined as the proportion of days an individual had access to the medication divided by the number of days during the period of interest. Proportion of days covered was calculated based on filled prescriptions during the follow-up period,^24^ except for aspirin for which the PDC period was set to 30 days. Per convention, adherence was defined as PDC > 80%, while a patient with PDC < 80% was considered nonadherent.^24,25^ Due to differences in dosing regimen, PDC for ticagrelor and prasugrel was calculated separately. Adherence to vitamin K antagonist (VKA) treatment is generally evaluated as the time in therapeutic range measured by international normalized ratio (INR). Unfortunately, the Danish registries do not contain complete INR data on all participants throughout the study period. Therefore, adherence to VKA treatment was reported as next filled prescription after index event.

### Outcomes

The primary efficacy outcome was major adverse cardiovascular events (MACE), defined as a composite of all-cause death, stroke, recurrent MI, or repeat revascularization at 12 months (see Supplementary material online, *Table S2*). The primary safety outcome was bleeding events requiring hospitalization. Bleeding events included cerebral, gastrointestinal, and urogenital bleeding.

Secondary outcomes included the individual components of MACE; a MACE outcome including cardiovascular death; the individual bleeding outcomes; net adverse clinical events (NACE), defined as death from any cause, stroke, recurrent MI, or bleeding events requiring hospitalization; and a MACE outcome without repeat revascularization defined as all-cause death, MI, or stroke.

To increase the probability of the outcomes being truly related to the exposure, we employed a falsification outcome analysis including an endpoint presumed unrelated to the treatment.^26^ The falsification endpoint was a composite of hospitalizations for falls, most common fractures in the age group (humeral, radial, fingers, and femoral), dehydration, and acute kidney injury (see Supplementary material online, *Table S2*).

A blanking period of 30 days was employed for all outcomes except stroke to ensure the outcomes were independent of the index event.

### Statistical methods

Continuous variables were presented as medians (first to third quartiles, Q1–Q3) and compared across groups with the Mann–Whitney *U* test. Categorical variables were shown as counts and percentages and compared across groups using Pearson’s χ^2^ test. Due to regulations of Statistics Denmark, absolute numbers of either 1 or 2 were reported as not applicable (NA).

Because of a low number of patients claiming a prescription for prasugrel, we combined patients treated with ticagrelor and prasugrel into one group. Therefore, the two study groups comprised a clopidogrel group and a ticagrelor or prasugrel group.

Absolute and relative risks for outcomes were estimated using multivariable logistic regression with average treatment effect modelling (G-formula).^27^ This method aims to create a more uniform comparison of treatment groups by equal distribution of factors with a potential impact on outcomes. The primary efficacy outcome was standardized to the distribution of the individual components of the CHA_2_DS_2_-VASc score [heart failure, hypertension, age, diabetes mellitus, prior stroke/transient ischaemic attack/venous thromboembolism, sex (Supplementary material online, *Table S1*)], type of OAC treatment (DOAC or VKA), and claimed prescriptions for aspirin. As a diagnosis of MI was a mandatory inclusion criterion, all patients scored at least 1 point in the vascular disease item of the CHA_2_DS_2_-VASc score. The primary safety outcome was standardized to the available, individual components of the HAS-BLED score [hypertension, renal disease, liver disease, stroke, prior bleeding requiring hospitalization, medication predisposing to bleeding (NSAID and aspirin), and excess alcohol use (Supplementary material online, *Table S1*)], sex, type of OAC, and concomitant treatment with PPI.

A sensitivity analysis with a χ^2^ test was performed to evaluate if a causal relationship between claim of a prescription for aspirin and P2Y_12_ inhibitor group existed. A two-sided *P*-value of <0.05 was considered statistically significant.

SAS version 9.4 (SAS institute, Inc., Cary, NC, USA) was used for data management and RStudio, version 4.0.3 (https://www.r-project.org/), for statistical analysis.

### Ethics

Data access and use of the Statistics Denmark server were approved by the appropriate data responsible unit in the Capital Region of Denmark (approval number *P*-2019-403).

## Results

### Patients and characteristics

Between 2011 and 2019, 2259 patients with AF treated with OAC were admitted for first-time MI, treated with PCI, and claimed a prescription for P2Y_12_ inhibitor after discharge. Of these, 1918 patients claimed a prescription for clopidogrel, 303 for ticagrelor, and 38 for prasugrel. Patients treated with ticagrelor or prasugrel were combined into 1 group comprising 341 individuals.

Median age was 74 years (Q1–Q3: 67–81) in the clopidogrel group and 70 years (Q1–Q3: 62–77) in the ticagrelor or prasugrel group. Women comprised 24.0% of the ticagrelor group and 29.2% of the ticagrelor or prasugrel group. Patients treated with clopidogrel were more likely to have known coronary artery disease, hypertension, and had a higher CHA_2_DS_2_-VASc score. Population characteristics are summarized in *Table 1*. Concomitant PPI treatment was also more likely in the clopidogrel group. Prescription medications at discharge are summarized in *Table 1*.

**Table 1:** Baseline demographics, comorbidities, and post-discharge medication stratified by treatment group

	Clopidogrel (*n* = 1918)	Ticagrelor or prasugrel (*n* = 341)	*P*-value
Age, years [median (Q1–Q3)]	74 (67, 81)	70 (62, 77)	<0.001
Women	560 (29.2%)	82 (24.0%)	0.06
Coronary artery disease	184 (9.6%)	19 (5.6%)	0.02
Peripheral artery disease	128 (6.7%)	16 (4.7%)	0.21
Hypertension	1186 (61.8%)	168 (49.3%)	<0.001
Diabetes	315 (16.4%)	55 (16.1%)	0.95
COPD	171 (8.9%)	32 (9.4%)	0.86
Heart failure	156 (8.1%)	31 (9.1%)	0.63
Ischaemic stroke	119 (6.2%)	15 (4.4%)	0.24
Chronic kidney disease	44 (2.3%)	4 (1.2%)	0.26
Cancer	162 (8.4%)	25 (7.3%)	0.56
CHA_2_DS_2_-VASc score [median (IQR)]	4 (3, 5)	3 (2, 4)	<0.001
HAS-BLED score [median (IQR)]	2 (1, 3)	2 (1, 2)	0.001
Direct oral anticoagulant	1137 (59.3)	100 (29.3)	<0.001
Aspirin	1370 (71.4%)	259 (76.0%)	0.09
PPI	918 (47.9%)	116 (34.0%)	<0.001

COPD, chronic obstructive pulmonary disease; IQR, interquartile range; PPI, proton pump inhibitor.

### Primary efficacy outcome

**Figure 2:**
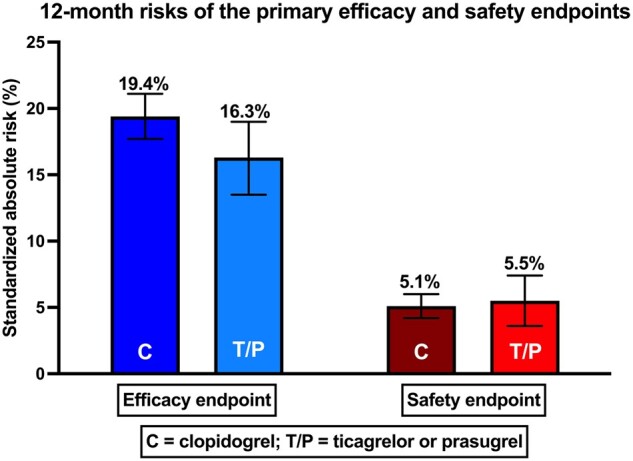
Standardized absolute risk of primary efficacy (major adverse cardiovascular events) and safety (bleeding events requiring hospitalizations) outcomes at 12 months stratified by treatment group (clopidogrel vs. ticagrelor or prasugrel). C, clopidogrel; T/P, ticagrelor/prasugrel.

**Figure 3:**
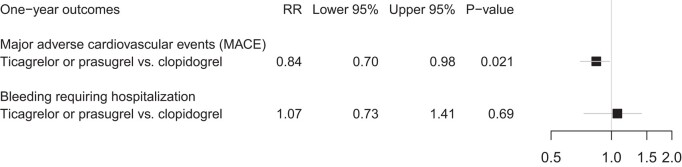
Standardized relative risk of primary efficacy (major adverse cardiovascular events) and safety (bleeding events requiring hospitalizations) outcomes at 12 months comparing the two treatment groups (ticagrelor or prasugrel vs. clopidogrel).

Standardized absolute risks of MACE at 12 months were 19.4% [95% confidence interval (CI), 17.8–21.1] for clopidogrel and 16.3% (95% CI, 13.5–19.0) for ticagrelor or prasugrel (*Figure 2*). The standardized relative risk was 0.84 (95% CI 0.70–0.98, *P* = 0.02) for ticagrelor or prasugrel vs. clopidogrel (*Figure 3*).

### Primary safety outcome

The standardized absolute risks of bleeding were 5.1% (95% CI, 4.2–6.0) for clopidogrel and 5.5% (95% CI 3.6–7.4) for ticagrelor or prasugrel, respectively (*Figure 2*). The standardized relative risk was 1.07 (95% CI 0.73–1.41, *P* = 0.69) for ticagrelor or prasugrel vs. clopidogrel (*Figure 3*).

### Falsification outcome

The standardized absolute risks of the composite of common causes of hospitalization were 4.1 (95% CI 3.2–5.0) for clopidogrel and 4.6 (95% CI 2.1–7.2) for ticagrelor or prasugrel. The standardized relative risk was 1.10 (95% CI 0.47–1.80, *P* = 0.67) for ticagrelor or prasugrel vs. clopidogrel (see Supplementary material online, *Figure S1*).

### Interaction

No significant interaction was detected between claim of a prescription for aspirin and P2Y_12_ inhibitor group for either of the primary outcomes (*P* = 0.79 for the efficacy outcome, *P* = 0.28 for the safety outcome).

### Adherence

In the clopidogrel group, 1452 patients (75.7%) had a PDC > 80%. Moreover, 124 (40.9%) in the ticagrelor group and 31 (81.6%) in the prasugrel group had a PDC > 80%. The low adherence in the ticagrelor group was a result of de-escalation. During the follow-up period, 138 individuals initially treated with ticagrelor later claimed a prescription for clopidogrel (38 switched during the first 90 days, 34 between 91 and 180 days, and 66 between 181 days and 1 year).

Due to the relatively low adherence in the ticagrelor group, we performed the primary efficacy and safety analyses on a subgroup consisting only of patients with high adherence (PDC > 80%). The standardized absolute risk of MACE in the high adherence subgroup analysis was 14.5% (95% CI, 12.7–16.2) for the clopidogrel group and 11.2% (95% CI, 8.3–14.1) for the ticagrelor or prasugrel group (see Supplementary material online, *Figure S2*). The relative risk of MACE was 0.77 (95% CI, 0.58–0.97, *P* = 0.02). The absolute risks of bleeding events requiring hospitalizations were 4.4% (95% CI, 3.4–5.4) and 3.8% (95% CI, 1.8–5.8) in the clopidogrel and ticagrelor or prasugrel group, respectively. The relative risk of bleeding events was 0.86 (95% CI, 0.44–1.28, *P* = 0.53) (see Supplementary material online, *Figure S2*).

Individuals receiving DOAC and aspirin generally had a high PDC (average >90%). Supplementary material online, *Table S3* summarizes the frequency of PDC > 80% for the individual DOAC drugs and aspirin. Of the 1022 patients in the VKA group, 918 claimed a second prescription during the follow-up period. The risks of MACE and bleeding events requiring hospitalization were calculated for two subgroups of patients on triple therapy: one group consisting of individuals on DOAC therapy with PDC > 80% for both DOAC and aspirin and one group consisting of individuals claiming a second prescription for VKA and with PDC > 80% for aspirin. For the subgroup of individuals on triple therapy including DOAC, the absolute risk of MACE was 16.1% (95% CI 12.7–19.4) for the clopidogrel group and 16.7% (95% CI 8.3–25.2) for the ticagrelor or prasugrel group. The relative risk of MACE was 1.0 (95% CI 0.52–1.60, *P* = 0.87). The corresponding absolute bleeding risk was 4.0% (95% CI 2.2–5.9) and 1.9% (95% CI 0.0–4.7) for clopidogrel and ticagrelor or prasugrel, respectively (see Supplementary material online, *Table S4*). The relative risk of bleeding events was 0.49 (95% CI 0–1.19, *P* = 0.15) The standardized absolute risk of MACE in the triple therapy group including VKA was 19.9% (95% CI 15.6–24.1) in the clopidogrel group and 16.0% (95% CI 9.4–22.6) in the ticagrelor or prasugrel group, and the absolute risks of bleeding were 4.9% (95% CI 2.5–7.2) and 5.5% (95% CI 1.2–9.7), respectively (see Supplementary material online, Table S4). The relative risk of MACE with ticagrelor or prasugrel vs. clopidogrel was 0.81 (95% CI 0.48–1.10, *P* = 0.24), and the relative risk of bleeding events was 1.10 (95% CI 0.24–2.01, *P* = 0.79).

### Secondary outcomes

**Figure 4:**
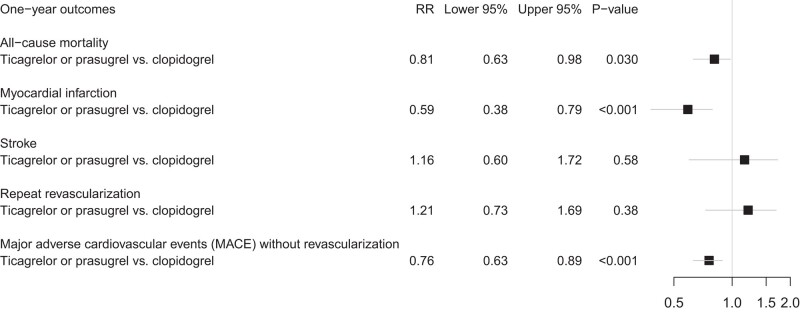
Standardized relative risk of the secondary outcomes comparing the two treatment groups (ticagrelor or prasugrel vs. clopidogrel).

The standardized absolute risk of death from any cause was 8.3% (95% CI, 7.1–9.5) in the clopidogrel group and 6.7% (95% CI, 5.1–8.3) in the ticagrelor or prasugrel group. The corresponding standardized relative risk was 0.81 (95% CI 0.63–0.98, *P* = 0.03) for ticagrelor or prasugrel vs. clopidogrel. A forest plot of the standardized relative risks of all secondary outcomes is presented in *Figure 4*.

## Discussion

In this nationwide, registry-based study of patients with a history of AF admitted with a first-time MI treated with PCI and who filled a prescription for a P2Y_12_ inhibitor after discharge, we found that treatment with ticagrelor or prasugrel was associated with a reduced risk of MACE, without a concomitantly increased risk of bleeding when compared with clopidogrel.

In the Dual Antithrombotic Therapy with Dabigatran after PCI in Atrial Fibrillation (RE-DUAL PCI) trial as well as in the Antithrombotic Therapy after Acute Coronary Syndrome or PCI in Atrial Fibrillation (AUGUSTUS) trial, clopidogrel was the recommended P2Y_12_ inhibitor for patients with coronary artery disease treated with PCI, but the treating physician was allowed to prescribe a more potent P2Y_12_ inhibitor if deemed indicated. Contrary to our findings, subgroup analyses of AUGUSTUS and RE-DUAL PCI reported that treatment with a potent P2Y_12_ inhibitor was associated with an increased bleeding risk but no ischaemic benefit.^28,29^ In fact, subjects receiving ticagrelor in the RE-DUAL PCI trial had a numerically higher risk of MACE (18.7% vs. 12.9%). These results are reflected in contemporary European guidelines that recommend clopidogrel as the P2Y_12_ inhibitor of choice in patients with ACS and an indication for concomitant OAC undergoing PCI.^12^

Major bleeding in patients with ACS is independently associated with a five-fold increase in the risk of death,^30^ and bleeding risk appears more prominent in AF patients with ACS, irrespectively of antithrombotic regimen.^31^ The fact that we did not find a difference in bleeding between the two treatment groups may be attributable to our definition of bleeding events that only included bleeding requiring hospitalizations. The Danish registries do not currently include complete data on haemoglobin levels or bleeding diagnoses made at general practitioners’ clinics.^32^ Accordingly, it is unlikely that minor bleeding events were captured. Furthermore, individuals in the clopidogrel group were more likely to be treated with a PPI. These agents have previously been associated with a lower bleeding risk when administered alongside dual antiplatelet therapy.^33,34^ Although we standardized our analyses to the distribution of PPI treatment, we cannot exclude the possibility that the neutral relative risk in terms of bleeding across treatment groups was driven by a higher baseline bleeding risk in the clopidogrel group.

The randomized trials executed in this field focused on triple vs. double therapy and comparison of DOACs with VKAs.^15–18^ Collectively, the greatest reduction in bleeding risk were achieved by de-escalating from triple to double therapy. On the other hand, dual antiplatelet therapy with aspirin and a P2Y_12_ inhibitor has been considered crucial to reduce the risk of in-stent stenosis in non-AF subjects undergoing PCI.^35,36^ In an attempt to balance the risk of recurrent ischaemia with risk of bleeding, guidelines recommend most AF patients to receive a short period of triple therapy.^12^ In our population, it was more likely for patients in the potent P2Y_12_ inhibitor group to claim a prescription for aspirin. This supports the notion that patients at a higher perceived ischaemic risk were preferentially prescribed ticagrelor or prasugrel. However, in Denmark, aspirin can be purchased as over-the-counter medication which is not captured in the Danish National Prescription Register possibly leading to underestimation of aspirin use in our population.

A German, registry-based study compared prasugrel with clopidogrel in 377 patients requiring triple therapy.^37^ The investigators found an increased bleeding risk but no ischaemic advantage of prasugrel. Patients treated with prasugrel had a higher baseline risk profile compared with those treated with clopidogrel, and the entire study population was on triple therapy. Given the overlap between risk factors for thrombosis and bleeding,^38,39^ it is possible that in our setting, patients with an overall higher baseline risk profile were preferentially prescribed clopidogrel. Despite attempts to account for the differences in baseline risk, results might have been skewed in favour of the potent P2Y_12_ inhibitors.

Finally, a Canadian, prospective, observational study comparing ticagrelor with clopidogrel in 277 patients with AF and MI treated with PCI on triple therapy found no differences in MACE or bleeding risk, irrespectively of the type of P2Y_12_ inhibitor.^40^ Considering the totality of evidence, the clear preference of clopidogrel over the more potent P2Y_12_ inhibitors may be questionable.

### Limitations

Some imbalances in baseline patient characteristics are inevitable in observational studies. The European (and thus, Danish) guidelines on AF patients with ACS undergoing PCI recommends clopidogrel as the P2Y_12_ inhibitor of choice.^12,41^ The use of ticagrelor or prasugrel is considered an active choice by the treating physician. Employing average treatment effect modelling is an attempt to minimize the effect of confounding by indication. Due to the specific setting of our study, the sample was small, particularly with respect to patients receiving prasugrel, thus limiting the generalizability of our results to this drug. Combining the ticagrelor and the prasugrel group may have impacted the results as these medications are not entirely interchangeable.^42^ Clopidogrel is metabolized by the cytochrome P450 2C19 (CYP2C19) enzyme system to the active drug, and according to the US Food and Drug Administration, 2–14% of the population are so-called poor metabolizers of clopidogrel due to CYP2C19 genetic variation.^43^ It is not yet common practice in Denmark to perform CYP2C19 genotyping, and thus, reduced metabolism could have impacted our results in favour of ticagrelor and prasugrel. On the other hand, studies investigating choice of P2Y_12_ inhibitor guided by platelet reactivity have not been able to definitively demonstrate improved outcomes. This is also reflected in contemporary guidelines which do not recommend genotyping in routine clinical practice.^44,45^ Moreover, our primary efficacy outcome included repeat revascularization. It is contemporary practice to defer treatment of secondary lesions in ACS patients with multivessel disease.^46^ Despite our use of a 30-day blanking period, it is possible that some staged events related to the index MI were included, though this is unlikely to have affected our primary efficacy endpoint as there was no between-group difference in repeat revascularization. Furthermore, individuals in the ticagrelor group exhibited a lower adherence than both the clopidogrel and prasugrel groups. The lower adherence was due to de-escalation of P2Y_12_ inhibitor which might have impacted the results. Nevertheless, our results were confirmed in the subgroup analysis including only individuals with high adherence, and it is thus unlikely that lower adherence in one group impacted the overall results. Guideline recommendations on the use of aspirin in patients requiring concomitant OAC and platelet inhibition have evolved over the years. Despite this, both OAC and aspirin treatment was characterized by a high adherence rate. Although limited by small sample sizes and low overall event rates, the subgroup analyses of patients with high adherence to triple therapy yielded results that were overall consistent with the main results. It was more frequent for patients in the clopidogrel group to claim a prescription for a PPI, but overall, the rate of PPI use was lower than what might have been expected for such a study population. The fact that PPIs in small packages are sold over the counter in Denmark may have resulted in underreporting of the number of patients on PPI. Lastly, we did not have information on body weight, complexity of coronary artery disease, stent type, or true drug adherence.

## Conclusions

In this nationwide, retrospective, registry-based study of patients with AF on OAC who underwent PCI for MI, individuals prescribed ticagrelor or prasugrel had a lower risk of ischaemic events and death, but not an increased risk of bleeding, than those who were prescribed clopidogrel. While these findings appear to support an individualized choice of P2Y_12_ inhibitor in this population, the results should ideally be confirmed in a randomized clinical trial.

## Supplementary Material

oead134_Supplementary_Data

## Data Availability

Data access and use of the Statistics Denmark server were approved by the appropriate data responsible unit in the Capital Region of Denmark (approval number P-2019-403).
