# Supplementary material for: Ticagrelor or prasugrel vs. clopidogrel in patients with atrial fibrillation undergoing percutaneous coronary intervention for myocardial infarction
Source: Eur Heart J Open. 2023 Dec 14;4(1):oead134. doi: 10.1093/ehjopen/oead134 (PMC10763543; doi:10.1093/ehjopen/oead134)

One−year outcomes	RR	Lower 95%	Upper 95%	P−value
Major adverse cardiovascular events (MACE)				
Ticagrelor or prasugrel vs. clopidogrel	0.77	0.58	0.97	0.022
Bleeding requiring hospitalization				
Ticagrelor or prasugrel vs. clopidogrel	0.86	0.44	1.29	0.53

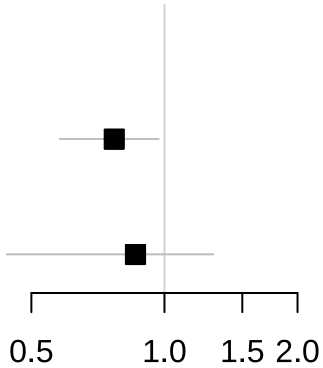

Supplement: oead134_Supplementary_Data [file oead134_supplementary_data.zip › Supplementary Figure S2.pdf]
