# Supplementary material for: Ticagrelor or prasugrel vs. clopidogrel in patients with atrial fibrillation undergoing percutaneous coronary intervention for myocardial infarction
Source: Eur Heart J Open. 2023 Dec 14;4(1):oead134. doi: 10.1093/ehjopen/oead134 (PMC10763543; doi:10.1093/ehjopen/oead134)
Supplement: oead134_Supplementary_Data [file oead134_supplementary_data.zip › Supplementary table S1+S2+S3+S4.docx]

**Table S1:** Definitions of inclusion and comorbidity diagnoses, procedure codes and medication. Abbreviations: ICD: International Classification of Diseases 10^th^ revision, NOMESCO: Nordic Medico Statistical Committee classification of procedures, ATC: Anatomical Therapeutic Chemical code, COPD: chronic obstructive pulmonary disease, TIA: transitory ischemic attack, VTE: venous thromboembolism,

| **Diagnosis/definition** | **ICD** | **NOMESCO** | **ATC** |
| --- | --- | --- | --- |
| Atrial fibrillation | DI48* |  | B01AF01, B01AF02, B01AF03, B01AF07, B01AA0*, B01AA03 |
| Myocardial infarction | DI200, DI201* |  |  |
| Percutaneous coronary intervention |  | KFNF, KFNG |  |
| Venous thromboembolism | DI26, DI80, DI81 |  |  |
| Diabetes mellitus | DE10, DE11, DE12, DE13, DE14 |  | A10* |
| COPD | DJ40, DJ41, DJ42, DJ43, DJ44 |  | R03BB, R03AL |
| Hypertension | DI10, DI11, DI12, DI13, DI15 |  | C0* |
| Chronic kidney disease | DN18, DN19, DE102, DE112, DE142, DI120 |  |  |
| Heart failure | DI50, DI42, DI11, DI13 |  |  |
| Ischemic stroke | DI61, DI63, DI64 |  |  |
| Cancer | DC* |  |  |
| Anemia | DD5*, DD60, DD61, DD62, DD63, DD64 |  |  |
| Defect of coagulation cascade | DD65, DD66, DD67, DD68, DD69 |  |  |
| Liver disease | DD7* |  |  |
| Peripheral artery disease | DI70, DI71, DI72, DI73 |  |  |
| Coronary artery disease | DI201, DI208, DI209, DI24, DI25 |  |  |
| CHA_2_DS_2_-VASc | **Heart failure**: DI50, DI42, DI11, DI13; **hypertension**: DI10, DI11, DI12, DI13, DI15; d**iabetes**: DE10, DE11, DE12, DE13, DE14; **stroke/TIA/VTE**: DI61, DI63, DI64, DG458, DG459, DI26, DI80, DI81; |  | **Antihypertensives**: C0*; **anti-diabetics**: A10* |
| HAS-BLED | **Hypertension**: DI10, DI11, DI12, DI13, DI15; **chronic kidney disease**: DN18, DN19, DE102, DE112, DE142, DI120; **liver disease**: DD7*, **stroke**: DI61, DI63, DI64; **bleeding events**: DI60, DI61, DI62, DN02, DR31, DN938, DN939, DN950, DN421, DN838F, DI850, DK250, DK252, DK254, DK256, DK260, DK262, DK264, DK266, DK270, DK272, DK274, DK276, DK80, DK282, DK284, DK286, DK290, DK920, DK921, DK922, DK625, DK661, DK638B, DK638C, DN838F; **alcohol**: DF10, DK70, DE52, DT51, DK860, DE244, DG312, DI426, DO354, DZ714, DZ721, DG621, DG721, DK292, DL278A, DE8609 |  | **Aspirin**: B01AC06;  **Non-steroidal anti-inflammatory drugs**: M01A |

**Table S2**: Definitions of outcomes in terms of diagnoses and procedure codes. Abbreviations: ICD: International Classification of Diseases 10^th^ revision, NOMESCO: Nordic Medico Statistical Committee classification of procedures

| **Outcome** | **ICD** | **NOMESCO** |
| --- | --- | --- |
| Myocardial infarction | DI200, DI21*, DT823D, DT823E |  |
| Stroke | DI61, DI63, DI64 |  |
| Revascularization |  | KFNA, KFNB, KFNC, KFND, KFNE, KFNF, KFNG |
| Bleeding events (gastrointestinal, cerebral, urogenital) | DI60, DI61, DI62, DN02, DR31, DN938, DN939, DN950, DN421, DN838F, DI850, DK250, DK252, DK254, DK256, DK260, DK262, DK264, DK266, DK270, DK272, DK274, DK276, DK80, DK282, DK284, DK286, DK290, DK920, DK921, DK922, DK625, DK661, DK638B, DK638C, DN838F |  |
| Falsification outcome: falls, most common fractures, dehydration, and acute kidney failure | DN178, DN179, DR296, DS42, DS52, DS62, DS72, DE86, DN19 |  |

**Table S3:** Frequency of individuals with PDC >80% versus total group count (regardless of P2Y_12_ inhibitor) for the individual direct oral anticoagulants as well as aspirin.

| **Drug** | **PDC >80% vs total group count, n =** |
| --- | --- |
| Apixaban | 361/463 |
| Rivaroxaban | 399/518 |
| Dabigatran | 155/238 |
| Edoxaban | 10/14 |
| Aspirin | 1011/1014 |

**Table S4**: Triple therapy subgroup sizes and summary of standardized absolute risks of major adverse cardiovascular events and bleeding events requiring hospitalization.

| **Subgroup** |  | **Clopidogrel** | **Ticagrelor or prasugrel** |
| --- | --- | --- | --- |
|  |  |  |  |
| Direct oral anticoagulant + aspirin + P2Y_12_ inhibitor |  | n = 429 | n = 34 |
|  | Major adverse cardiovascular events | 16.1% (95% CI 12.7-19.4) | 16.7% (95% CI 8.1-25.2) |
|  | Bleeding events requiring hospitalization | 4.0% (95% CI 2.2-5.9) | 1.9% (95% CI 0.0-4.7) |
|  |  |  |  |
| Vitamin K antagonist + aspirin + P2Y_12_ inhibitor |  | n = 312 | n = 51 |
|  | Major adverse cardiovascular events | 19.9 % (95% CI 15.6-24.1) | 16.0% (95% CI 9.4-22.6) |
|  | Bleeding events requiring hospitalization | 4.9% (95% CI 2.5-7.2) | 5.5% (95% CI 1.2-9.7) |
