# Supplementary material for: Ticagrelor or prasugrel vs. clopidogrel in patients with atrial fibrillation undergoing percutaneous coronary intervention for myocardial infarction
Source: Eur Heart J Open. 2023 Dec 14;4(1):oead134. doi: 10.1093/ehjopen/oead134 (PMC10763543; doi:10.1093/ehjopen/oead134)

One−year outcomes	RR	Lower 95%	Upper 95%	P−value
Hospitalization for falls, fractures, dehydration, or acute kidney injury				
Ticagrelor or prasugrel vs. clopidogrel	1.10	0.47	1.80	0.67

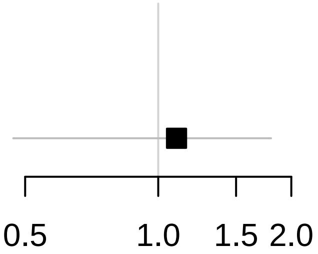

Supplement: oead134_Supplementary_Data [file oead134_supplementary_data.zip › Supplementary Figure S1.pdf]
